# Have We Looked in the Wrong Direction for More Than 100 Years? Delayed Onset Muscle Soreness Is, in Fact, Neural Microdamage Rather Than Muscle Damage

**DOI:** 10.3390/antiox9030212

**Published:** 2020-03-05

**Authors:** Balazs Sonkodi, Istvan Berkes, Erika Koltai

**Affiliations:** Department of Health Sciences and Sport Medicine, University of Physical Education, 1123 Budapest, Hungary; berkesdr@gmail.com (I.B.); koltai.erika@tf.hu (E.K.)

**Keywords:** DOMS, superposition of compression, muscle spindle, acute compression axonopathy, gate control, closed gate exercise, mitochondrial, free radicals

## Abstract

According to our hypothesis, delayed onset muscle soreness (DOMS) is an acute compression axonopathy of the nerve endings in the muscle spindle. It is caused by the superposition of compression when repetitive eccentric contractions are executed under cognitive demand. The acute compression axonopathy could coincide with microinjury of the surrounding tissues and is enhanced by immune-mediated inflammation. DOMS is masked by sympathetic nervous system activity at initiation, but once it subsides, a safety mode comes into play to prevent further injury. DOMS becomes manifest when the microinjured non-nociceptive sensory fibers of the muscle spindle stop inhibiting the effects of the microinjured, hyperexcited nociceptive sensory fibers, therefore providing the ‘open gate’ in the dorsal horn to hyperalgesia. Reactive oxygen species and nitric oxide play a cross-talking role in the parallel, interlinked degeneration–regeneration mechanisms of these injured tissues. We propose that the mitochondrial electron transport chain generated free radical involvement in the acute compression axonopathy. ‘Closed gate exercises’ could be of nonpharmacological therapeutic importance, because they reduce neuropathic pain in addition to having an anti-inflammatory effect. Finally, DOMS could have an important ontogenetical role by not just enhancing ability to escape danger to survive in the wild, but also triggering muscle growth.

## 1. Introduction

Delayed onset muscle soreness (DOMS) has been defined as delayed onset soreness, muscle stiffness, swelling, loss of force-generating capacity, reduced joint range of motion, and decreased proprioceptive function [1]. Theodore Hough wrote about DOMS first in 1902 associating the soreness with ruptures in the muscles [2], but the mechanism of DOMS is still not entirely understood. In DOMS, the pain is not felt for about 8 h, peaks 1 or 2 days later [3], and subsides within 7 days after exercise [4]. The pain is thought to be a result of microtrauma to the muscles followed by inflammation [4]. Several theories, such as lactic acid, muscle spasm, inflammation, connective tissue damage, muscle damage, and enzyme efflux, try to explain the mechanism of DOMS [5], but no single theory has answered the problem entirely. 

According to our *superposition of compression with cognitive demand-induced acute axonopathy* theory, the injury of the entrapped excessively compressed nerve endings in the muscle spindle could be rather the prevailing cause of the soreness and other symptoms. The same force could damage the muscle as a coinciding phenomenon, which further enhances, but does not cause the symptoms of DOMS [4]. 

## 2. Background of our Hypothesis

### 2.1. The Muscle Spindle

Colon et al. [6] formulated a detailed description of the muscle spindle. Muscle spindles contain intrafusal muscle fibers with sensory, gamma-motoneuron (γ-MN) [7,8,9], and sympathetic innervation [10]. Their main function is proprioception, and through this information, an internal representation of the body’s position and movement to the brain is provided, meaning also the global sense of ourselves [11]. “Upon a change in muscle tension, the muscle spindles send signals through afferent sensory neurons which are relayed to neurons within the spinal cord. Motoneurons receiving afferent information can then signal intrafusal or extrafusal fibers to relax or contract in response to sensory input [7]. With the afferent sensory feedback, the reflex arc acts as an automated closed loop so that voluntary movement can be achieved accurately and properly” [6]. 

**Figure 1:**
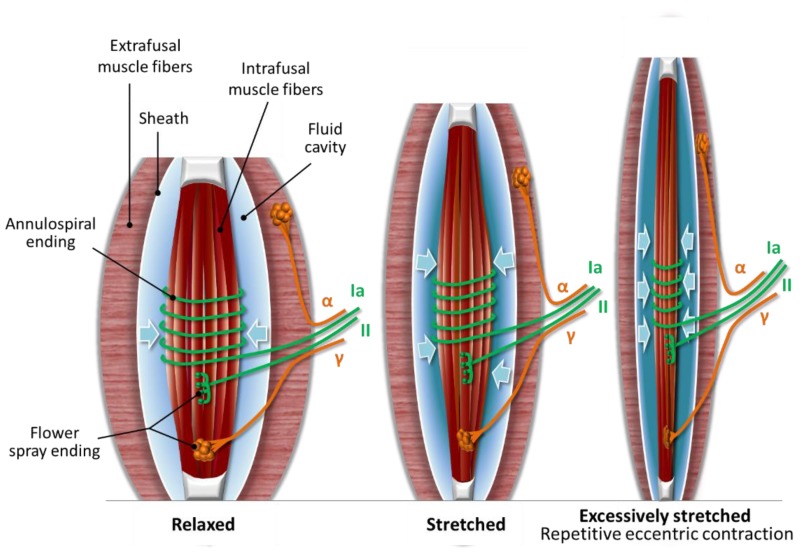
Three positions of the muscle spindle. (1) Relaxed muscle spindle: the more relaxed the muscle spindle is, the more relaxed the fluid cavity becomes; (2) Stretched muscle spindle: when the muscle spindle stretches, the fluid cavity flattens with uncompressible fluid inside, resulting in more compression and firing of the nerve terminals; (3) Excessively stretched muscle spindle: uncompressible fluid entraps and causes microinjury to the nerve terminals due to the superposition of compression when repetitive eccentric exercise is executed in an unaccustomed or strenuous way (drawn based on Colon’s description [6]).

The sensory innervation of intrafusal fibers consists of Type Ia and Type II sensory neurons. “The Type Ia sensory neurons innervate the intrafusal fibers with annulospiral wrappings around the equatorial region and Type II sensory neurons employ flower spray endings toward the peripheral ends of the fiber. γ-MNs innervate the peripheral ends of the intrafusal fibers via flower-spray endings as well (see Figure 1). The peripheral ends of the intrafusal fibers slowly relax or contract under the control of γ-MNs. These γ-MNs modulate the tension, sensitivity, and length of the intrafusal fibers so muscle spindles can maintain constant sensitivity during dynamic muscle action and prevent overextension, which can lead to undue stress on the muscle as well as tendons and cause joint damage [8]” [6]. In addition to the above, there is anatomical evidence of direct sympathetic innervation in human muscle spindles as well [10]. 

The outer capsule of the muscle spindle consists of flattened perineurial cells and a fluid-filled cavity could be found in the periaxial space. The perineurium of the afferent and efferent nerves of the muscle spindle is continuous with the outer capsule and serves as a selective barrier, such as the blood–brain barrier. The motor and sensory nerves are unmyelinated within the capsule of the muscle spindle [12]. Our theory attributes a functional role to the existence of the fluid cavity. In normal mode, the muscle spindle could be in three positions: stretched, relaxed (see the Stretched and Relaxed muscle spindle in Figure 1), or slack position, when only the extrafusal fibers contract. According to our hypothesis, when the muscle spindle stretches, the fluid cavity flattens with uncompressible fluid inside, resulting in more firing in the Type Ia sensory and Type II sensory fiber nerve endings, due to compression of this sandwich position (see the Stretched muscle spindle in Figure 1). The more relaxed the muscle spindle is, the more relaxed the fluid cavity becomes, meaning less firing in the sensory nerve terminals due to less compression. In the slack position, there is no stimulus on the sensory nerve terminals.

### 2.2. Compression Microinjury of the Nerve Endings in the Muscle Spindle

Bewick et al. highlighted that stretch induced deformation in the sensory region of the nerve terminals in frog muscle spindle [13]. It has been also observed that stretch of the mammalian muscle spindle comes along with the extension of the sensory region and increased spacing between the annulospirals of the primary-ending terminals [13,14,15]. 

In eccentric exercise, we propose that the muscle spindle is excessively lengthened and the fluid cavity flattens out even more with uncompressible fluid inside, resulting in significantly enhanced compression (see the Excessively stretched muscle spindle in Figure 1). The enhanced compression could entrap the nerve endings within the muscle spindle, and the repetition of eccentric contractions could lead to microinjury of the nerve terminals. 

## 3. Hypothesis: DOMS is an Acute Compression Axonopathy with Possible Coinciding Tissue Microinjuries and Is Enhanced by Immune-Mediated Inflammation

Hody et al. [16] divided DOMS into two phases, where the initial mechanical damage is followed by a more severe secondary damage and cited Morgan et al. [17], who defined these phases: primary and secondary damage phases. We would not call the secondary phase a damage phase, even though we agree that some mechanisms involved are damaging, but it is rather about restoring homeostasis after the initial damage. 

### 3.1. Primary Damage Phase: Acute Compression Axonopathy and Possible Coinciding Tissue Microinjury during Eccentric Exercise

DOMS is usually felt after unaccustomed or strenuous exercise. At rest and moderate exercise, there is mainly parasympathetic control, while going into high intensities, there is a balanced shift toward sympathetic control [18,19]. Increased blood flow in proportion to the exercise intensity happens in parallel to increased sympathetic neural discharge to the active muscles [20]. Sympathetic stimuli to active muscles are increasing significantly even at very low forces and rising in proportion to the contraction force without threshold [21]. Since tendons and fasciae encapsulate the muscles into a compartment, we suspect that over time, this frontier with the increased blood flow and strong muscle force inside could induce a compartment effect with increased compression. Therefore, we view that the increasing sympathetic nervous system (SNS) activity during unaccustomed or strenuous exercise is an essential underlying factor in DOMS.

In addition to the above, if the muscle is excessively lengthened in eccentric exercise, we suspect that the nerves, muscle fibers, connective tissues and the muscle spindle will be even under a tunnel effect, causing further compression. According to the superposition principle [22] of physics, it is the superposition of compression that results in even higher compression force. Therefore, our theory entails that the superposition of compression—that is, eccentric lengthening in addition to sympathetic tone—implies a significantly higher force that could potentially cause microinjury to the affected muscle spindles, muscle fibers, and connective tissues, but the SNS activity-derived compression at high intensity exercise alone is not enough damaging force to reach the muscle spindle.

Beyond the above physically burdening situation, muscles are fatiguing during intense exercise. According to our theory, when unaccustomed or strenuous eccentric exercise is executed to a point when muscle is fatiguing and not capable of sufficient force production, but the muscle activity should be maintained cognitively at the previously accustomed performance level or accomplish a goal, then “going over the limit” is desired in order to accomplish. We propose that SNS activity in the form of an acute stress response (ASR) is such a homeostatic driver, which serves the purpose of “going over the limit” with direct sympathetic innervation in the muscle spindles. It has been demonstrated that once the fatiguing muscle cannot satisfy the force generating demand from intrinsic changes, then the excitability of the motoneuron pool could be facilitated by the afferent feedback [23,24]. Brownstone et al. [25] theorized that the facilitation of the premotor circuits on γ-MNs would contract the intrafusal muscle fibers out of proportion. Furthermore, focusing attention on a task secondary to any perceived weakness also increases muscle spindle sensitivity [26]. It has been shown that sustained submaximal isometric contraction ultimately leads to a progressive decline in the discharge of muscle spindle afferent [27]. According to the theory of Brownstone et al. [25], the increased premotor activity and the concomitant spindle afferent input will eventually end in an escape from homeostasis in amylotrophic lateral sclerosis. We propose that the above excessive coinciding mechanical and metabolic insults impair the energy supply of the mitochondria in the sensory terminals of the muscle spindle, and this energy deficiency eventually leads to an escape from homeostasis, which we call DOMS. 

The fluid cavity could behave similar to a switch in the initiation of DOMS, when the superposition of compression force reaches the level that microinjures the entrapped nerve terminals and compresses the nerve endings against the uncompressible fluid. Neuropathic pain formulation could be initiated due to the microinjury of the Type II sensory fiber terminals, and the fusimotor system will provide the reduced function of the neuromuscular reflex arc in order to protect from macroinjury [8]. The reduced function could be a result of the enhanced stimuli of the microinjured Type Ia sensory nerve terminals and γ-MNs of the fusimotor system, resulting in a decreased range of motion and eventually decreased muscle strength. The fine programming of the decreased range of motion and decreased muscle strength is accomplished by the cross-talking between Type Ia sensory fibers and γ-MNs, Type Ia sensory fibers and Type II sensory fibers, and also among the γ-, β-, and α-MNs. Based on this hypothesis, DOMS is rather an acute compression axonopathy than a muscle problem. 

In summary, we propose that in the primary damage phase of DOMS, the neuropathic pain formulation has been initiated due to neuronal microinjury, but it is not felt yet, and the reduced function of the fusimotor system on the motoneural reflex arc is not yet manifest, due to the suppressing effect of SNS activity. ASR could prevail on the basis of SNS activity in the primary damage phase as a homeostatic driver in order to sustain muscle force, which has two pathways in this process. The direct sympathetic innervation of the muscle spindle could increase sympathetic stimuli, which dampens the feedback control of muscle length [10,28,29,30], meaning that fine movements can be traded for “flight and fight” response [10]. SNS activity could also suppress pain by descending inhibition of nociception in the spinal cord [31], providing the “heat of battle” response. It is important to note that the descending inhibition of nociception in the spinal cord is suppressing the muscle pain caused by the Type III/IV muscle afferents in the fatiguing muscle [32], but not the delayed onset neuropathic pain, which is initiated but not felt yet in the primary damage phase. According to the Gate Control Theory of Pain [33], the faster conducting, microinjured non-nociceptive fibers (Type Ia sensory fibers) indirectly inhibit the effects of microinjured nociceptive fibers (Type II sensory fibers) by closing the gate to the transmission of pain stimuli [34]. We propose that ASR could happen during unaccustomed or strenuous eccentric exercise when muscles are fatiguing and in this short ASR time window in the primary damage phase when repetitive lengthening contractions could microdamage the nerve endings in the muscle spindle.

According to our hypothesis, the *superposition of compression with cognitive demand-induced acute axonopathy* inside the muscle spindle is essential in DOMS. The possible microinjury by the same superposition of compression force of the surrounding muscles and connective tissues is a coinciding and later interlinked phenomenon, but it is not a must for DOMS [4].

### 3.2. Secondary Phase: Microinjury-Induced Immune-Mediated Inflammation and Regeneration after Microdamaging Eccentric Exercise

It is not the subject of this paper to explain the theories of DOMS or judge them, but rather to highlight those scientific findings that are in support of our hypothesis. Our theory entails that several parallel immune-mediated degeneration–regeneration processes could be in the secondary phase of DOMS in the affected microinjured tissues, such as the muscles and connective tissues. These parallel degeneration–regeneration and concomitant inflammation processes could be tissue-specific and not necessarily overlapping in terms of timeline, but they could be interlinked through cross-talking. Tissue injuries and inflammation are coupled to facilitate pain sensation [35], but they are not essential to DOMS [4], except for the microinjury of the sensory neurons of the muscle spindle. The immune and nociceptive systems are destined to identify noxious stimuli, and as a result, they are triggering responses to prevent tissue damage and restoring homeostasis [35]. 

Once unaccustomed or strenuous exercise is finished and SNS activity subsides, then immune-mediated inflammation and their cross-talking at multiple interfaces comes into play in the microinjured tissues until regeneration. Examples for the interfaces of cross-talking are the cyclooxygenase-1 (COX-1)–prostaglandin E2 (PGE2) pathway; the COX-2–nerve growth factor (NGF) pathway; the COX-2, PGE2, and glial cell line-derived neurotrophic factor (GDNF) pathway, leukotrienes; cytokines; reactive oxygen species; and nitric oxide. 

#### 3.2.1. Mechanical Hyperalgesia: Neuropathic Pain Enhanced by Inflammatory Pain

Lund et al. demonstrated that primary afferent neurons in the muscle spindle could relate to allodynia [36]. Injury of the nociceptive neurons could trigger molecular changes and as a result develop pathological spontaneous activity [37]. Under pathological condition, large-sized primary sensory neurons may become hyperexcitable, which is mediated by COX-1–PGE2 pathway [38]. 

According to our theory, the pain stimuli could arise in the primary damage phase of DOMS due to pathological condition—that is the compressed, microinjured Type II fiber nerve endings, causing hyperexcitability. This could be the initiation of the buildup of neuropathic pain, but the microdamaged, faster conducting non-nociceptive fibers (Type Ia sensory fibers) could indirectly inhibit the effects of nociceptive fibers (Type II sensory fibers) by closing the gate to the transmission of their stimuli, resulting in a hypoalgesic state based on the Gate Control Theory of Pain [34]. This indirect inhibition could be the reason for the delay of pain sensation in the secondary phase of DOMS.

Zhu et al. found in neuropathic rats that only the injured non-nociceptive neurons in low-threshold mechanoreceptors, similar to the muscle spindle, showed significantly reduced conduction velocity with a delayed onset, and they suspected that these neurons are related to allodynia [39]. Lund et al. demonstrated the masseter muscle spindle Ia afferents involvement in the development of allodynia in rats and also found small-caliber nociceptive axons aside from the primary afferents [36]. The findings of Zhu et al. also showed that C-fiber dorsal root ganglion neurons are likely not the primary sensory neurons causing tactile allodynia, but rather A-type sensory neurons [39,40,41].

According to our *superposition of compression with cognitive demand-induced acute axonopathy* theory, the non-nociceptive, Type Ia sensory axon terminals could go through pathological changes in the microinjured annulospiral nerve endings, and this axonopathy could result in a significantly slower conduction velocity with a delayed onset [39]; therefore, they become incapable of indirectly inhibiting the effects of the hyperexcited, nociceptive Type II sensory fibers. Thus, the axonopathy of Type Ia sensory neurons are providing exclusively the ‘open gate’ and the pathway to DOMS. Furthermore, we would not exclude the role of the direct sympathetic innervation in this velocity coupling. Schlereth et al. [31] raised the possibility that SNS could control peripheral inflammation and nociceptive activation. Since SNS is capable of reversing spinal descending inhibition to spinal facilitation [31], it is likely that inflammatory muscle pain from Type IV or C-fibers could be facilitated by SNS once there is an ‘open gate’ resulting in the coupling of neuropathic and inflammatory pain pathways in DOMS.

The delayed onset of pain is typically felt at muscle stretch and contraction but not at rest [42]. According to our theory, there are no stimuli on Type II sensory terminals in the relaxed position of the muscle spindle (see the Relaxed muscle spindle in Figure 1), but once stretch and contraction arises, then there is stimuli on the hyperexcited Type II sensory terminals (see the Stretched or Excessively stretched muscle spindle in Figure 1). This theory could also explain that soreness is caused by eccentric exercise and isometric exercise at a lesser extent due to the magnitude of microinjury causing the excessive stretch elements of these exercises (see the Excessively stretched muscle spindle in Figure 1). Pure concentric exercise causes no soreness [42], because excessive stretch elements are minimal or non-existent, therefore causing no superposition of compression on the nociceptive terminals (see the Stretched muscle spindle in Figure 1). 

Pinho et al. [35] noted that tissue injury and inflammation are interlinked processes resulting in elevated pain sensitivity (hyperalgesia) in DOMS. The increased sensitivity of nociceptor activity is a result of PGE2 by stimulating ion channels [43]. Mizumura et al. [4] reviewed that NGF is produced when ischemia [44] or nerve injury [45] happens in DOMS, but the COX-2–GDNF pathway is also involved with cross-talking at the COX-2 level [46]. Murase et al. [47] showed in rats that bradykinin release during exercise plays an essential role in triggering muscular mechanical hyperalgesia. Nociceptors in the surrounding microinjured tissues could be also stimulated, thereby further enhancing pain sensation [5]. Findings are even showing that the elevation of pain sensitivity was greater for fascia than for muscle [48,49,50]. 

According to our theory, mechanical hyperalgesia could be a result of an acute compression axonopathy (primary damage phase) enhanced by inflammatory pain (secondary phase). Pain in DOMS has been attributed to C-fibers from the microinjured tissues [4], but we propose that the axonopathy of the microinjured Type Ia sensory fibers in the muscle spindle could provide exclusively the ‘open gate’ to pain in DOMS. Therefore, the hyperexcited, microinjured Type II sensory neurons could propagate pain earlier to ‘open gate’. C-fibers involvement in hyperalgesia could be only secondary, but important, because these neurons contribute to slow temporal summation [34]. The coupling of the pain pathways could be facilitated by SNS activity [31] once the gate is open. The ‘open gate’ means central nervous system (CNS) involvement and control on the dorsal horn [34], but this important modulation of CNS is not the subject of this paper. 

In summary, based on Sun et al. [38], we propose that the COX-1–PGE2 pathway could sensitize Type II sensory fibers in the microdamaged muscle spindle. Based on Mizumura et al. [4], the COX-2–PGE2–GDNF pathway could sensitize Type III sensory fibers, and based on Murase et al. [47], the COX-2–NGF pathway could sensitize Type IV sensory or C-fibers in the microdamaged muscle. Murase et al. [46] presented that there is a cross-talk on the level of COX-2. According to our theory, this cross-talk could mean that the pain pathways of Type III and C-fibers are interlinked after muscle microdamage (inflammatory pain pathway), if there is any. We also propose, if there is coinciding muscle damage with DOMS, then there could be cross-talk on the PGE2 level, resulting in the coupling of the pain pathways of Type II sensory fibers (neuropathic pain) with the already interlinked Type III/IV sensory fibers. Furthermore, our theory identifies the COX-1–PGE2 pathway as a critical pathway in DOMS. The COX-1–PGE could be the mechanism that Mizumura et al. [4] suspected to be present in DOMS, beyond identifying the muscle fiber damage and inflammation (COX-2–PGE2–GDNF and COX-2–NGF pathways) not essential for DOMS.

#### 3.2.2. Immune-Mediated Inflammation

Cheung et al. [5] reviewed that repetitive eccentric muscle contractions will result in a buildup of edema and inflammatory cell infiltration [51,52,53]. The proteolytic enzymes in muscle fibers start to degrade the injured cell structures. This breakdown process with concomitant elevated bradykinin, histamine, and prostaglandins invites monocytes and neutrophils to the injury site [54]. An exercise-induced increased permeability of microcirculation will gradually result in elevated protein-rich fluid in the muscle [53]. Eventually, the increasing osmotic pressure induces compression [55], leading to a compartment effect on sensory nerves and on the affected tissues.

Murase et al. [47] demonstrated that the upregulation of NGF mRNA was also present in DOMS in a 12 h to 2 d timeframe, and that is correlating with the delayed onset peak of muscle soreness in DOMS [3]. The peak edema level in the damaged tissues also appears to coincide with peak muscle soreness [56,57]. 

Proteases and phospholipases are activated by calcium accumulation after sarcolemma damage with concomitant leukotrienes and prostaglandins production [58,59]. Leukotrienes increase vascular permeability, in addition to attracting neutrophils [60]. The firstly arriving and activated neutrophils will feed the inflammatory cycle by phagocytosis, releasing oxygen free radicals and proteases and causing further tissue injury [61]. 

Mizumura et al. [4] reported that muscle fiber damage and inflammation are not essential for DOMS, and other mechanisms must be present [4]. There is evidence in human study of no differences between inflammatory markers for individuals who executed eccentric contraction and those who executed concentric contraction [62]. Another study showed the existence of mechanical hyperalgesia without muscle injury in electrically stimulated muscle undergoing eccentric contractions [63]. 

These findings are in line with our hypothesis that the cause of the initiation of DOMS could be the *superposition of compression with cognitive demand-induced acute axonopathy* in the muscle spindle, and the inflammation of the microinjured tissues, including the muscle, plays a secondary, but not casual role in the secondary phase by facilitating the symptoms of DOMS. 

Kuphal et al. [64] showed in animal studies that swimming (concentric exercise) is a nonpharmacological approach for the management of peripheral neuropathic pain. Our *superposition of compression with cognitive demand-induced acute axonopathy theory* could be of importance in using exercise intervention as a therapeutic tool in neuropathic pain and disease management, because by keeping a ‘closed gate’ with exercise, the neuropathic pain could be reduced, but all the beneficial anti-inflammatory characteristics of exercise could be enjoyed. Therefore, we propose the use of ‘closed gate exercise’ and ‘open gate exercise’ terms from a therapeutic point of view. ‘Closed gate exercise’ would mean the type of exercises that are not microinjuring the non-nocicpetive primary afferent of the muscle spindle. In contrary, ‘open gate exercise’ could be a stimulus on nociceptive neurons, because the micorinjured primary afferent neurons are not capable of inhibiting them; therefore, it is associated with DOMS. 

It has been demonstrated on rats that even a remote injury on a peripheral nerve could result in a leakage of the blood–spinal cord barrier (BSCB) through a selective inflammatory pathway [65]. Beggs et al. [66] showed that peripheral nerve injury and electrical stimulation of C-fibers each cause a transient increase in BSCB and blood–brain barrier (BBB) permeability. The increase of BSCB permeability could not be observed 6 h after the injury of the nerve, but was apparent after 24 h, peaked at about 24–48 h, and returned to normal level 7 days after peripheral nerve injury [66]. Not surprisingly, the timeline of this transient BSCB permeability [66] is highly correlating with the timeline of DOMS [3,4]. Another study involving mouse demonstrated T-cell infiltration and activation in the dorsal horn of the spinal cord after peripheral nerve injury, contributing to the buildup of neuropathic pain-like hypersensitivity [67]. Radu et al. [68] proposed that T-cell infiltration in the nerve-injured animals may be correlating with the increase in BSCB permeability. Libby et al. [69] unsuspectedly revealed that inflammatory signaling networks at work in ischemic cardiovascular diseases linked local and systemic inflammation. According to our theory, we propose that a similar leakage of the BSCB after ‘open gate exercise’ is executed with a resultant inflammatory cross-talking. We argue that ‘open gate exercise’ could potentially link local inflammation (DOMS) to systemic inflammation (aging, neurogenerative diseases, autoimmune diseases, etc.). Furthermore, we propose a leakage during DOMS at the selective barrier of the muscle spindle [12] as well. Without this leakage, the proposed cross-talk on the PGE2 level could not happen.

#### 3.2.3. Decrease in Muscle Strength and Stiffness

Decreases in muscle power and a reduced range of motion are characteristic symptoms of DOMS [5,70,71]. These decreases have been devoted to the microdamage of the injured muscle fibers [72], but DOMS does not always come with muscle damage [63,73]. On the other hand, stiffness, as an another symptom of DOMS, has been believed to be related to inflammatory cause rather than to muscle damage [74]. 

According to our hypothesis, we propose that these symptoms are primarily a result of the safety function of the motoneural reflex arc caused by the acute compression axonopathy of the non-nociceptive primary afferent neuron in the muscle spindle. The muscle damage and inflammation are only secondary and are not exclusive to DOMS [4]. The safety function of the motoneural reflex arc could serve the purpose of avoiding macroinjury [8] when the fatigued muscle’s α-MNs receives less input from the microinjured non-nociceptive primary afferent neurons. We propose that in ontogenesis, the annulospiral nerve endings of the primary afferent neurons evolved into this higher surface ending form with a purpose, other than sensing propriception, to be more prone to microinjury in repetitive excessive lengthening situations. When microdamage happens, the non-nociceptive neurons show significantly reduced conduction velocity with a delayed onset [39] and therefore less input on α-MNs, resulting in the safety function of the motoneural reflex arc in the form of decreased muscle strength and stiffness.

## 4. Reactive Oxygen Species (ROS) and Nitric Oxide (NO)

The involvement of ROS in DOMS has been reported [75], and the source of production is attributed to inflammatory agents in the muscle [76]. Free radicals play a crucial role in the degeneration process of removing the damaged muscle. In addition, they are also important in the regeneration process as signaling molecules to regulate muscle cell growth, differentiation, and proliferation [77].

Radak et al. were the first to propose the involvement of NO in DOMS [78,79] with functions such as decreased muscle strength [80,81], pain sensation, and regeneration [82,83]. According to our theory, we expect similar involvement of NO in the secondary phase of DOMS. 

Cashman et al. [84] emphasized the enhanced vulnerability of the nervous system to free radical damage because of the high energetic demand [85]. Nitric oxide has a protective vasodilatory effect, but its radical could damage proteins, lipids, and cells [86], likely resulting in energetic failure [87] and apoptosis [88,89]. The result of these derangements includes increased nociception as well as the distal degeneration of nerve fibers [90].

We propose that mitochondrial electron transport chain-generated free radicals should not be excluded in the acute compression sensory axonopathy of DOMS. According to our theory, the force due to the superposition of compression under cognitive demand could possibly cause such a severe metabolic insult on axonal mitochondria in the sensory neurons of the muscle spindles that impairs the axon’s energy supply, which is similar to Bennett et al.’s [90] hypothesis explaining terminal arbor degeneration (TAD). According to Bennett et al., “if the energy deficiency is severe enough then degeneration happens, and the threshold for degeneration will be lowest in the neuronal compartment that has the highest energy requirement [90]”. They propose that the sensory axon’s terminal arbor is the compartment with the highest energy requirement. We believe that the muscle spindle is an analog compartment in DOMS. 

Holland et al. [91] already proposed the possibility of “terminal axonopathy” in 1998, which is an idea that is close to the TAD concept. An increased number of mitochondria could be found in such a location where the metabolic demand is high [92,93] and the sensory terminals of the muscle spindle are abundant with mitochondria [12]. Bennet et al. [90] also demonstrated that paclitaxel, which is an axonopathy-causing antineoplastic agent, evoked mechano-allodynia and mechano-hyperalgesia with a delayed onset. The appearance of delayed onset symptoms was threshold driven by accumulating toxicity and dosage dependent. The neuropathic pain induced by paclitaxel did not cause degeneration to the axon of the peripheral nerve, but TAD lesion alone could have been sufficient to produce mechano-hyperalgesia. We suspect a similar lesion on the nerve terminals of the sensory neurons in the muscle spindle that causes the mechano-hyperalgesia in DOMS.

## 5. DOMS and Ontogenesis

Elefteriou et al. [94] reviewed how the rapid proliferation of calcitonin gene-related peptide (CGRP) positive sensory nerves are involved in new bone growth in deer antlers [95]. Evidence of neuronal involvement was supported by the findings that bone growth reduced after denervation [96,97], supporting the functional role of sensory neurons in bone growth. The rapid proliferation of a periosteal dense network of CGRP- and substance P positive sensory nerves is also observed in rodents in fracture repair [98,99,100]. The periosteum is primarily innervated by this highly dense network of CGRP-positive sensory fibers that are sensitive to nociceptive stimuli [101] and pain [102], but sympathetic fibers are also innervating this bone compartment [103], as in the muscle spindle [10].

According to the hypothesis of Berger et al. [104], bones evolved ontogenetically to enhance the ability to escape danger in the wild. In line with this theory, they showed in animals and humans that stressors induce a rapid outflow of circulating osteocalcin, which is necessary to develop an ASR. Osteocalcin inhibits the activity of post-synaptic parasympathetic neurons in order to let the sympathetic tone become full blown [104]. Our theory of *superposition of compression with cognitive demand-induced acute axonopathy* entails that strenuous or unaccustomed eccentric exercise induced SNS activity could be an essential underlying factor at DOMS initiation with likely ASR involvement, so the possible role of osteocalcin in cross-talking should be investigated.

We also propose that beyond Berger et al.’s hypothesis of an enhanced ability to escape danger and thus survive in the wild, DOMS could have a significant role to play in ontogenesis by triggering nerves and surrounding tissues, such as causing muscles to grow and also by adapting the nervous system to comply with the growth process. The applicability of Hilton’s rule [94] indirectly implies that the muscles and bones possibly grow hand in hand in an analogue process. We propose that sensory nerve guidance in the muscle spindle could have a similar functional and essential role in muscle growth, similar to what has been observed in bone growth.

## 6. Conclusions

According to our hypothesis, DOMS is an acute compression axonopathy of the nerve terminals in the muscle spindle caused by the repetitive superposition of compression with a coinciding cognitive demand, coupled with possible microinjury to the surrounding tissues and enhanced by immune-mediated inflammation. Our theory states that DOMS happens only if the superposition of compression reaches the muscle spindle and microinjures the nerve terminals under cognitive demand. The cornerstones of our hypothesis are as follows:

- DOMS could be an acute compression axonopathy of the nerve endings in the muscle spindle,
- The cause of DOMS could be the repetitive superposition of compression under cognitive demand and a resultant metabolic insult,
- DOMS could be initiated from the muscle spindle,
- The fluid cavity in the muscle spindle could play an important functional role in DOMS,
- Mitochondrial electron transport chain generated free radical involvement is suspected with a TAD-like lesion in the acute axonopathy of the sensory nerve endings in DOMS,
- Unaccustomed or strenuous eccentric exercise-induced SNS activity could be an essential underlying factor in DOMS initiation,
- DOMS could be initiated earlier than it is experienced, but at the beginning, it is suppressed by SNS activity,
- Delayed onset of soreness could be a result of the hypoalgesic state of the ‘closed gate’ caused by the enhanced firing of the microinjured Type Ia sensory fibers in addition to the initial SNS suppression,
- There could be a cross-talk on the PGE2 level between the pain pathways,
- Hyperexcited microinjured Type II sensory fibers in the muscle spindle could override, with the possible help of SNS, the conduction velocity reducing microinjured Type Ia sensory fibers’ inhibition with a delayed onset. The result will be an ‘open gate’ in the dorsal horn and the pathway to hyperalgesia in DOMS,
- Keeping a ‘closed gate’ with concentric exercise could have importance in non-pharmacological disease and neuropathic pain management by simultaneously alleviating pain and enjoying the positive anti-inflammatory characteristics of exercise; therefore, we call it a ‘closed gate exercise’,
- DOMS could cause a transient increase of the blood–spinal cord barrier and selective muscle spindle barrier permeability,
- DOMS could be a safety function in repetitive eccentric contractions as it resolves when the microinjury of the muscle spindle afferent sensory and motoneuron nerve endings are regenerated,
- Finally, we suspect that DOMS could play an important role in ontogenesis by triggering muscle growth and adapting the nervous system in the growth process.

The variability of timelines and symptoms of DOMS, after initiation, will depend on how pervasive the injury is to the surrounding tissues and what tissues are affected. The individual differences could be explained by the complexity of pathways and cross-talking of the microinjured tissues and immune systems. The type and duration of eccentric exercise, trained status, age, genetics, and underlying allergies and low-grade inflammation or diseases could also affect the timelines and the extent of symptoms in DOMS.
